# Early postoperative fever as a predictor of pancreatic fistula after pancreaticoduodenectomy: a single-center retrospective observational study

**DOI:** 10.1186/s12893-024-02521-0

**Published:** 2024-08-12

**Authors:** Jae-Woo Ju, Hwan Suk Jang, Mirang Lee, Ho-Jin Lee, Wooil Kwon, Jin-Young Jang

**Affiliations:** 1 Department of Anesthesiology and Pain Medicine, Seoul National University Hospital, 101 Daehak-ro, Jongno-gu, Seoul, 03080 Republic of Korea; 2 Department of Anesthesiology and Pain Medicine, Seoul National University College of Medicine, Seoul, Republic of Korea; 3 Department of Surgery, Asan Medical Center, Seoul, Republic of Korea; 4 Department of Surgery, Seoul National University Hospital, Seoul, Republic of Korea; 5 Department of Surgery, Seoul National University College of Medicine, Seoul, Republic of Korea

**Keywords:** Fever, Pancreaticoduodenectomy, Pancreatic fistula, Pancreatic neoplasms, Postoperative complications

## Abstract

**Background:**

The connection between early postoperative fever and clinically relevant postoperative pancreatic fistula (CR-POPF) after pancreaticoduodenectomy remains unclear. This study aimed to investigate this association and assess the predictive value of early postoperative fever for CR-POPF.

**Methods:**

This retrospective observational study included adult patients who underwent pancreaticoduodenectomy at a tertiary teaching hospital between 2007 and 2019. Patients were categorized into those with early postoperative fever (≥ 38 °C in the first 48 h after surgery) and those without early postoperative fever groups. Weighted logistic regression analysis using stabilized inverse probability of treatment weighting (sIPTW) and multivariable logistic analysis were performed. The c-statistics of the receiver operating characteristic curves were calculated to evaluate the impact on the predictive power of adding early postoperative fever to previously identified predictors of CR-POPF.

**Results:**

Of the 1997 patients analyzed, 909 (45.1%) developed early postoperative fever. The overall incidence of CR-POPF among all the patients was 14.3%, with an incidence of 19.5% in the early postoperative fever group and 9.9% in the group without early postoperative fever. Early postoperative fever was significantly associated with a higher risk of CR-POPF after sIPTW (adjusted odds ratio [OR], 1.73; 95% confidence interval [CI], 1.34–2.22; *P* < 0.001) and multivariable logistic regression analysis (adjusted OR, 1.88; 95% CI, 1.42–2.49; *P* < 0.001). The c-statistics for the models with and without early postoperative fever were 0.76 (95% CI, 0.73–0.79) and 0.75 (95% CI, 0.72–0.78), respectively, showing a significant difference between the two (difference, 0.02; 95% CI, 0.00–0.03; DeLong’s test, *P* = 0.005).

**Conclusions:**

Early postoperative fever is a significant but not highly discriminative predictor of CR-POPF after pancreaticoduodenectomy. However, its widespread occurrence limits its applicability as a predictive marker.

**Supplementary Information:**

The online version contains supplementary material available at 10.1186/s12893-024-02521-0.

## Background

Postoperative fever occurs frequently following surgery and is triggered by various infectious and non-infectious causes [1, 2]. Early postoperative fever, which develops within 48 h of surgery, more commonly originates from non-infectious causes [1, 3], such as inflammatory cytokine release due to surgical trauma [4]; however, in these cases, fever is likely to be self-limiting and subsides within days [5]. Therefore, early postoperative fever is not necessarily indicative of severe postoperative complications, and costly laboratory analysis to investigate its causes may not be beneficial [6].

Postoperative pancreatic fistula (POPF) is the primary complication associated with pancreaticoduodenectomy, with clinically relevant POPF (CR-POPF), as defined by the International Study Group on Pancreatic Fistula, occurring in approximately 15% of patients [7]. It results in prolonged hospital stay, increased medical costs, and increased mortality [8, 9]. Pancreatic juice leakage due to POPF can lead to inflammation and auto-destruction of peripancreatic and retroperitoneal tissues, which can cause peripancreatic and retroperitoneal fluid collection, hemorrhage, intra-abdominal abscess, and sepsis. POPF most commonly manifests as fever, possibly due to the triggering of local or systemic inflammatory responses [10, 11], and early postoperative fever after pancreaticoduodenectomy can, therefore, be an important sign of POPF. However, the association between early postoperative fever and POPF has not been examined.

We aimed to investigate the association between early postoperative fever and the occurrence of CR-POPF in patients who had undergone pancreaticoduodenectomy. Furthermore, we investigated whether early postoperative fever was predictive of CR-POPF when combined with previously established predictors of CR-POPF.

## Methods

### Study design and population

We conducted a retrospective observational study based on adult patients who underwent pancreaticoduodenectomy at our institution between January 2007 and December 2019. The study protocol was reviewed and approved by the Institutional Review Board (IRB) of Seoul National University Hospital (IRB No. H-2308-177-1461). The requirement for informed consent was waived by the IRB owing to the de-identified and anonymized nature of the data, which cannot be traced back to identify individual patients. This study was conducted in accordance with the Declaration of Helsinki and the Strengthening the Reporting of Observational Studies in Epidemiology guidelines [12].

We enrolled consecutive adults aged 18 years and older who underwent elective pancreaticoduodenectomy. A priori sample size calculation was not performed. We excluded patients with missing information regarding confounders, those who died within 7 days of the index surgery, those who underwent total pancreatectomy during the index hospitalization, those who underwent concomitant surgery including portal vein resection, and those with preoperative fever (defined as the last recorded body temperature > 38 °C).

### Data collection and exposure

The data used in this study were acquired by retrospectively by reviewing electronic medical records using the Seoul National University Hospital Patient Research Environment (SUPREME). We collected data regarding patient demographics (sex, age, body mass index [BMI], and smoking status), cancer-related variables (neoadjuvant chemotherapy, neoadjuvant radiotherapy, preoperative biliary drainage, and pathology), surgery-related variables (type of surgery [pylorus-preserving pancreatoduodenectomy or Whipple procedure], surgical approach [robot-assisted or open], use of trans-anastomotic pancreatic ductal stent, and year of surgery), and intraoperative data (estimated blood loss, fluid administration [colloid, crystalloid], and transfusion). Additionally, information regarding pancreatic texture, pancreatic duct diameter (mm), and incidence of postoperative complications such as POPF was obtained from the surgeon’s database. The fistula risk score (0–10 points), which is an index developed and validated for predicting CR-POPF [13, 14], was calculated using pancreatic gland texture, pathology, pancreatic duct diameter, and intraoperative blood loss.

Data on perioperative body temperature were also collected using the SUPREME. Core temperature was continuously monitored using esophageal thermometers during surgery. Postoperatively, tympanic thermometers were employed for regular temperature checks at 4-hour intervals by rounding nurses, who recorded the data in the electronic medical record. If abnormal values were noted in the body temperature or other vital signs were noted, the body temperature was measured more frequently at the discretion of the attending physician. Among the preoperative temperature measurements taken after admission, the measurement closest to the start of surgery was designated as the preoperative temperature value. Severe intraoperative hypothermia was defined as an intraoperative time-weighted average body temperature below 35 °C [15]. Early postoperative fever was diagnosed if any recorded body temperature exceeded 38 °C in the first 48 h following surgery. Patients were then categorized into two groups as follows: those with early postoperative fever and those without early postoperative fever. Delayed postoperative fever was diagnosed if the body temperature exceeded 38 °C for the first time between 48 h and 168 h following surgery.

The surgical procedure involved a pancreaticojejunal anastomosis using a two-layer, end-to-side, duct-to-mucosal technique, with optional inclusion of a trans-anastomotic pancreatic ductal stent [16]. Moxifloxacin was routinely administered intravenously within 60 min prior to the surgical incision as a prophylactic antibiotic, and tazobactam was administered in cases where cholangitis was identified before the operation. Surgical drains were strategically placed near the anastomotic site and typically removed 3–5 days postoperatively. Amylase concentrations in both the serum and drainage fluid were measured regularly on postoperative days 1, 3, 5, 7, and 10, and contrast-enhanced computed tomography was performed 5–7 days following surgery to detect postoperative complications. If there was no evidence of leakage, the peripancreatic drains were removed.

### Outcomes

The primary outcome was the occurrence of CR-POPF grade B or C, as defined by the International Study Group on Pancreatic Fistula criteria [17]. This includes POPF with at least one of the following: increased amylase activity > 3 times the normal level; persisting peripancreatic drainage > 3 weeks; clinically relevant change in management of POPF including prolonged hospital or intensive care unit stay, or therapeutic agents for fistula management; percutaneous, endoscopic, angiographic, or surgical intervention for POPF; signs of infection related to POPF; and POPF-related organ failure or death. Secondary outcomes included any POPF, postoperative pulmonary complications, wound complications, any major postoperative complications, length of postoperative hospital stay, unplanned readmission within 30 days, and postoperative 1-year mortality. Wound complications were defined as wounds requiring aggressive dressing, repair, or delayed drain removal. Mortality data were retrieved from the Korean National Population Registry database. Other outcomes included postoperative infectious complications (infected POPF, intra-abdominal abscess, superficial/deep surgical site infection, pneumonia, phlebitis, urinary tract infection, and sepsis), bacterial growth on culture test, atelectasis, and postoperative serum C-reactive protein level.

### Statistical analysis

The primary outcome was compared between the two study groups using the stabilized inverse probability of treatment weighting (sIPTW) method to minimize selection bias [18]. The stabilized inverse probability weights were derived from the multivariable logistic regression model that predicted the probability of a given patient experiencing early postoperative fever based on the following covariates: male (vs. female), age (years), BMI (kg/m^2^), current smoker, neoadjuvant chemotherapy, neoadjuvant radiotherapy, preoperative biliary drainage, pathology, preoperative body temperature (℃), surgery type (pylorus-preserving pancreatoduodenectomy vs. Whipple procedure), surgical approach (robot-assisted vs. open), trans-anastomotic pancreatic ductal stent, fistula risk score, operation time (hours), estimated blood loss mL), intraoperative colloid (mL), intraoperative crystalloid (mL), intraoperative transfusion, severe intraoperative hypothermia, and year of surgery (2007–2010, 2011–2013, 2014–2016, or 2017–2019).The weights were calculated as the inverse of the probability of early postoperative fever in the patients in the early postoperative fever group and the inverse of (1 – the probability of early postoperative fever) for patients in the no early postoperative fever group. These weights were then stabilized by multiplying them by the proportion of patients in each group. In addition, extreme weights larger than the 99th percentile or smaller than the 1st percentile were truncated to the values at the 99th and 1st percentiles, respectively. An absolute standardized mean difference (ASD) of ≥ 0.1 was considered indicative of an unbalanced distribution between the groups before and after sIPTW. Following sIPTW, intergroup differences in the primary outcome are presented as odds ratios (OR) with 95% confidence intervals (CIs). Secondary outcomes were compared using the same approach; intergroup differences for continuous variables are presented as mean differences with 95% CI. To assess the robustness of our findings, the E-value was calculated to evaluate the magnitude of unmeasured confounding variables [19]. The E-value estimates the minimum strength of association that an unmeasured confounder would need to have with both exposure and outcome to explain the observed association between exposure and outcome.

We performed a multivariable logistic regression analysis of the primary outcome to verify the consistency of our findings. Univariable logistic regression analyses were performed for early postoperative fever and its covariates. Subsequently, all variables were entered into a multivariable logistic regression analysis without applying the variable selection method. The variance inflation factor was used to assess multicollinearity among the incorporated variables.

Two multivariable models were constructed to assess the discriminative performance of early postoperative fever. Model 1 comprised all covariates, whereas Model 2 incorporated all covariates and early postoperative fever. Receiver operating characteristic curves were generated for both models, and the c-statistic and corresponding 95% CI were calculated. The c-statistics of the two multivariable models were compared using DeLong’s test.

All statistical analyses were performed using R software version 4.0.0 (R Foundation for Statistical Computing, Vienna, Austria). A *P*-value of < 0.05 was considered to indicate a statistically significant difference.

## Results

**Fig. 1:**
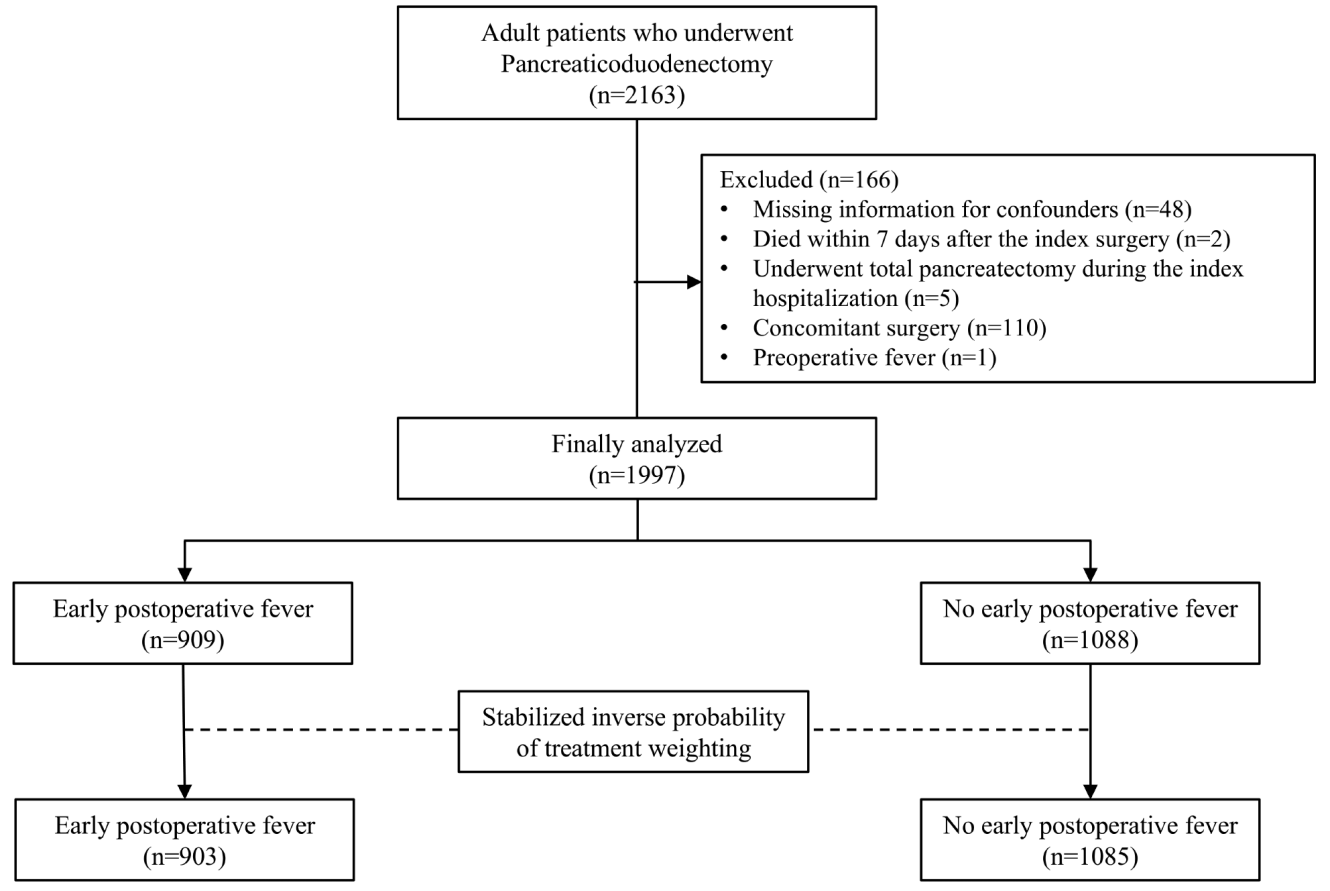
Flowchart of the study

**Fig. 2:**
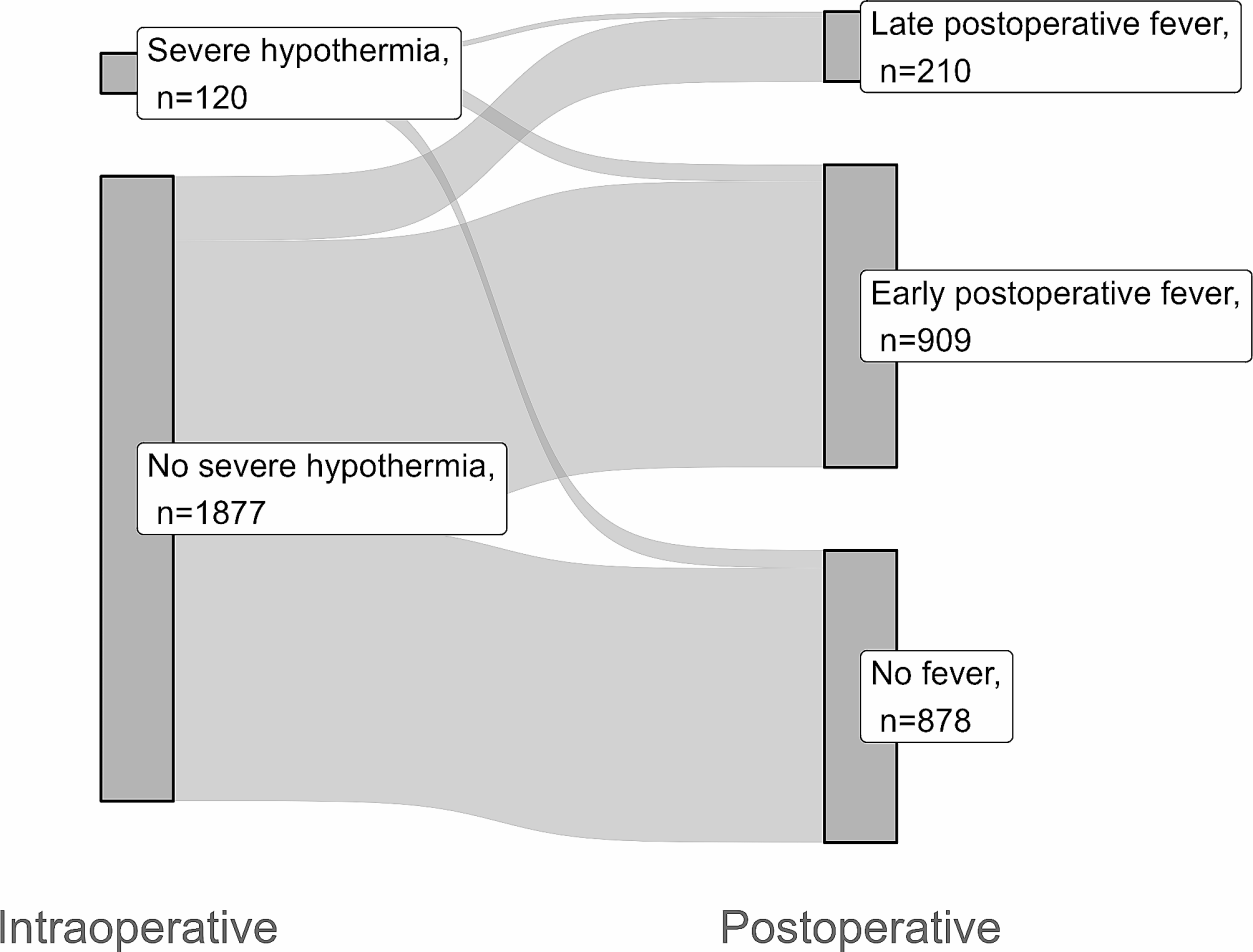
Sankey diagram showing the changes in body temperature during the perioperative period

Of the 2,163 patients who underwent pancreaticoduodenectomy, 166 (7.7%) were excluded based on the predefined exclusion criteria, as presented in Fig. 1. Consequently, 1,997 patients were analyzed, of which 909 (45.5%) experienced early postoperative fever and 1,088 (54.5%) did not. Figure 2 shows a Sankey diagram illustrating the changes in body temperature throughout the perioperative period.

Table 1 presents the baseline characteristics and surgical variables of patients in each group before and after sIPTW. Before sIPTW, patients in the early postoperative fever group were more likely to be male, older, current smokers, and have a higher BMI than those in the no early postoperative fever group. Following sIPTW, the two groups demonstrated well-balanced distributions, with all ASD values less than 0.1.

**Table 1:** Baseline characteristics and surgery-related variables between the study groups before and after sIPTW

	Before sIPTW					After sIPTW				
	EPF group (*n* = 909)	No EPF group (*n* = 1088)	ASD				EPF group (*n* = 903)	No EPF group (*n* = 1085)	ASD	
Male	600 (66.0%)	617 (56.7%)	0.192				552 (61.0%)	658 (60.6%)	0.009	
Age, years	63 (56–70)	65 (59–72)	0.240				64 (58–71)	65 (58–71)	0.006	
Body mass index, kg/m²	23.7 (21.6–25.7)	22.8 (21.0–24.8)	0.241				23.3 (21.2–25.4)	23.2 (21.3–25.1)	0.010	
Current smoker	155 (17.1%)	142 (13.1%)	0.112				132 (14.7%)	159 (14.6%)	0.001	
Neoadjuvant chemotherapy	67 (7.4%)	80 (7.4%)	0.001				65 (7.1%)	78 (7.2%)	0.003	
Neoadjuvant radiotherapy	31 (3.4%)	31 (2.8%)	0.032				28 (3.1%)	35 (3.2%)	0.003	
Preoperative biliary drainage	247 (27.2%)	253 (23.3%)	0.090				229 (25.3%)	272 (25.0%)	0.008	
Pathology			0.254						0.017	
Pancreatic cancer	315 (34.7%)	494 (45.4%)					360 (39.8%)	439 (40.4%)		
Distal CBD cancer	216 (23.8%)	189 (17.4%)					181 (20.1%)	216 (19.9%)		
Ampulla of Vater cancer	193 (21.2%)	184 (16.9%)					173 (19.2%)	206 (19.0%)		
Duodenal cancer	20 (2.2%)	32 (2.9%)					23 (2.5%)	28 (2.6%)		
Neuroendocrine tumor	72 (7.9%)	78 (7.2%)					68 (7.5%)	81 (7.5%)		
Benign diseases	53 (5.8%)	55 (5.1%)					52 (5.7%)	61 (5.7%)		
Others	40 (4.4%)	56 (5.1%)					46.5 (5.2%)	53.6 (4.9%)		
Preoperative temperature, ℃	36.5 (36.2–36.7)	36.4 (36.1–36.6)	0.113				36.4 (36.2–36.7)	36.4 (36.2–36.7)	0.011	
PPPD (vs. Whipple’s operation)	732 (80.5%)	805 (74.0%)	0.156				696 (77.1%)	833 (76.7%)	0.008	
Robot-assisted (vs. open)	107 (11.8%)	96 (8.8%)	0.097				93 (10.3%)	111 (10.2%)	0.003	
Trans-anastomotic pancreatic ductal stent	884 (97.2%)	1016 (93.4%)	0.184				863 (95.6%)	1033 (95.2%)	0.019	
Fistula risk score (0–10)	5 (3–6)	5 (3–6)	0.282				5 (3–6)	5 (3–6)	0.011	
Operation time, hour	5.2 (4.3–6.2)	5.2 (4.2–6.1)	0.086				5.2 (4.3–6.1)	5.2 (4.2–6.2)	< 0.001	
Estimated blood loss, mL	400 (250–600)	350 (200–500)	0.188				400 (250–550)	400 (250–550)	0.021	
Intraoperative colloid, mL	0 (0–500)	0 (0–500)	0.084				0 (0–500)	0 (0–500)	0.001	
Intraoperative crystalloid, mL	2600 (2000–3300)	2400 (1850–3100)	0.146				2500 (2000–3200)	2400 (1900–3200)	0.007	
Intraoperative transfusion	140 (15.4%)	135 (12.4%)	0.087				128 (14.2%)	152 (14.0%)	0.005	
Intraoperative severe hypothermia	50 (5.5%)	70 (6.4%)	0.039				55 (6.0%)	66 (6.1%)	0.002	
Year of surgery			0.159						0.009	
2007–2010	200 (22.0%)	259 (23.8%)					208 (23%)	252 (23.2%)		
2011–2013	205 (22.6%)	244 (22.4%)					199 (22%)	241 (22.2%)		
014–2016	179 (19.7%)	267 (24.5%)					208 (23%)	246 (22.7%)		
2017–2019	325 (35.8%)	318 (29.2%)					289 (32%)	346 (31.9%)		

The values are presented as the median (interquartile range) or numbers (proportion)

sIPTW, stabilized inverse probability of treatment weighting; EPF, early postoperative fever; ASD, absolute standardized mean difference; CBD, common bile duct; PPPD, pylorus-preserving pancreatoduodenectomy

The incidence of early postoperative fever was 62.1% (177/285) in patients who developed CR-POPF and 42.8% (732/1712) in those who did not. Primary and secondary outcome data before and after sIPTW are presented in Table 2. The overall incidence of CR-POPF was 14.3% (285/1997), with an incidence of 19.5% in the group with early postoperative fever and 9.9% in the group without early postoperative fever. Compared with patients in the no early postoperative fever group, those in the early postoperative fever group were at a significantly higher risk of CR-POPF in the weighted logistic regression analysis (adjusted OR, 1.73 95% CI, 1.34–2.22; *P* < 0.001). In addition, patients in the early postoperative fever group were at significantly increased risk of any pancreatic fistula (adjusted OR, 1.21; 95% CI, 1.01–1.45; *P* = 0.034), postoperative pulmonary complications (adjusted OR, 2.24; 95% CI, 1.33–4.10; *P* = 0.003), wound complications (adjusted OR, 2.79; 95% CI, 2.01–3.86; *P* < 0.001), major postoperative complications (adjusted OR, 2.17; 95% CI, 1.73–2.72; *P* < 0.001), and longer hospital stay (adjusted mean difference, 3.06 days; 95% CI, 1.79–4.33; *P* < 0.001). A comparison of other outcomes between the two groups is provided in **Supplementary Table ****S1****.**

**Table 2:** Comparison of the primary and secondary outcomes before and after sIPTW

	Before sIPTW				After sIPTW			
	EPF group (n = 909)	No EPF group (n = 1088)	OR or mean difference (95% CI)	P-value	EPF group (n = 903)	No EPF group (n = 1085)	Adjusted OR or mean difference (95% CI)	P-value
Clinically-relevant pancreatic fistula	177 (19.5%)	108 (9.9%)	2.19 (1.70–2.84)	< 0.001	161 (17.9%)	122 (11.2%)	1.73 (1.34–2.22)	< 0.001
Any pancreatic fistula	570 (62.7%)	586 (53.9%)	1.44 (1.20–1.72)	< 0.001	547 (60.6%)	606 (55.8%)	1.21 (1.01–1.45)	0.034
Postoperative pulmonary complications	34 (3.7%)	18 (1.7%)	2.31 (1.30–4.12)	0.004	36 (4.0%)	19 (1.8%)	2.34 (1.33–4.10)	0.003
Wound problems	125 (13.8%)	57 (5.2%)	2.88 (2.08–4.00)	< 0.001	123 (13.6%)	58 (5.3%)	2.79 (2.01–3.86)	< 0.001
Any major postoperative complications	248 (27.3%)	142 (13.1%)	2.50 (1.99–3.14)	< 0.001	238 (26.3%)	154 (14.2%)	2.17 (1.73–2.72)	< 0.001
Length of postoperative hospital stay, days	18.2 (18.4)	14.9 (8.3)	3.32 (2.10–4.54)	< 0.001	18.1 (18.2)	15.1 (8.5)	3.06 (1.79–4.33)	< 0.001
Unplanned readmission within 30 days	52 (5.8%)	59 (5.4%)	1.06 (0.72–1.56)	0.772	55 (6.1%)	64 (5.9%)	1.04 (0.72–1.51)	0.843
1-year mortality	88 (9.7%)	117 (10.8%)	0.89 (0.66–1.19)	0.432	96 (10.6%)	105 (9.7%)	1.10 (0.82–1.48)	0.513

The values are presented as the mean (standard deviation) or numbers (proportion)

sIPTW, stabilized inverse probability of treatment weighting; EPF, early postoperative fever; OR, odds ratio; CI, confidence interval

The E-value for the association between early postoperative fever and CR-POPF was 2.01. This indicates that there needs to be an associated unmeasured variable with an OR of at least 2.01 to render the significance of the association between early postoperative fever and CR-POPF as non-significant.

The results of the univariable and multivariable logistic regression analyses for the primary outcome are presented in Table 3. Multivariable analysis revealed that early postoperative fever was a significant risk factor for CR-POPF after pancreaticoduodenectomy (adjusted OR, 1.88; 95% CI, 1.42–2.49; *P* < 0.001). Male sex, higher BMI, distal common bile duct cancer, Ampulla of Vater cancer, neuroendocrine tumor, higher fistula risk score, and severe intraoperative hypothermia were also identified as risk factors for CR-POPF. Conversely, neoadjuvant chemotherapy and surgery in the later study period were associated with a lower risk of developing CR-POPF.

**Table 3:** Logistic regression analyses for clinically relevant pancreatic fistula after pancreaticoduodenectomy

	Univariable			Multivariable	
	Unadjusted OR (95% CI)	*P*-value		Adjusted OR (95% CI)	*P*-value
Early postoperative fever	2.19 (1.70–2.84)	< 0.001		1.88 (1.42–2.49)	< 0.001
Male (vs. female)	1.88 (1.42–2.49)	< 0.001		1.77 (1.29–2.43)	< 0.001
Age, years	1.00 (0.99–1.02)	0.452		1.01 (1.00–1.03)	0.079
Body mass index, kg/m²	1.09 (1.05–1.13)	< 0.001		1.09 (1.05–1.14)	< 0.001
Current smoker	1.05 (0.74–1.49)	0.772		0.85 (0.57–1.25)	0.406
Neoadjuvant chemotherapy	0.12 (0.04–0.37)	< 0.001		0.25 (0.06–1.07)	0.062
Neoadjuvant radiotherapy	0.10 (0.01–0.69)	0.020		0.81 (0.07–9.56)	0.866
Preoperative biliary drainage	1.23 (0.93–1.62)	0.155		1.21 (0.88–1.68)	0.240
Pathology					
Pancreatic cancer	Reference			Reference	
Distal CBD cancer	6.05 (4.19–8.73)	< 0.001		2.77 (1.78–4.31)	< 0.001
Ampulla of Vater cancer	3.19 (2.14–4.77)	< 0.001		1.67 (1.06–2.62)	0.027
Duodenal cancer	2.95 (1.31–6.62)	0.009		1.61 (0.67–3.87)	0.290
Neuroendocrine tumor	3.89 (2.35–6.41)	< 0.001		2.37 (1.36–4.11)	0.002
Benign diseases	2.61 (1.41–4.86)	0.002		1.66 (0.85–3.24)	0.135
Others	2.77 (1.46–5.24)	0.002		1.85 (0.93–3.72)	0.081
Preoperative temperature, ℃	0.72 (0.50–1.06)	0.096		0.88 (0.58–1.33)	0.539
PPPD (vs. Whipple’s operation)	1.52 (1.10–2.11)	0.012		1.10 (0.76–1.59)	0.621
Robot-assisted (vs. open)	0.79 (0.50–1.23)	0.294		1.08 (0.61–1.90)	0.795
Trans-anastomotic pancreatic ductal stent	2.20 (1.01–4.80)	0.047		1.86 (0.80–4.32)	0.150
Fistula risk score (0–10)	1.29 (1.21–1.37)	< 0.001		1.23 (1.12–1.34)	< 0.001
Operation time, hour	1.09 (0.99–1.19)	0.066		0.98 (0.86–1.12)	0.812
Estimated blood loss, every 100 mL	1.01 (0.99–1.03)	0.548		0.97 (0.93–1.01)	0.130
Intraoperative colloid, every 100 mL	1.04 (1.00–1.08)	0.031		1.00 (0.95–1.04)	0.852
Intraoperative crystalloid, every 100 mL	1.01 (1.00–1.02)	0.042		1.01 (0.99–1.03)	0.211
Intraoperative transfusion	0.92 (0.64–1.34)	0.677		0.84 (0.52–1.36)	0.485
Intraoperative severe hypothermia	1.64 (1.03–2.59)	0.036		1.74 (1.04–2.92)	0.036
Year of surgery					
2007–2010	Reference			Reference	
2011–2013	0.97 (0.70–1.35)	0.869		0.97 (0.67–1.41)	0.888
2014–2016	0.40 (0.27–0.59)	< 0.001		0.33 (0.21–0.51)	< 0.001
2017–2019	0.42 (0.30–0.59)	< 0.001		0.34 (0.21–0.53)	< 0.001

OR, odds ratio; CI, confidence interval; CBD, common bile duct; PPPD, pylorus-preserving pancreatoduodenectomy

**Fig. 3:**
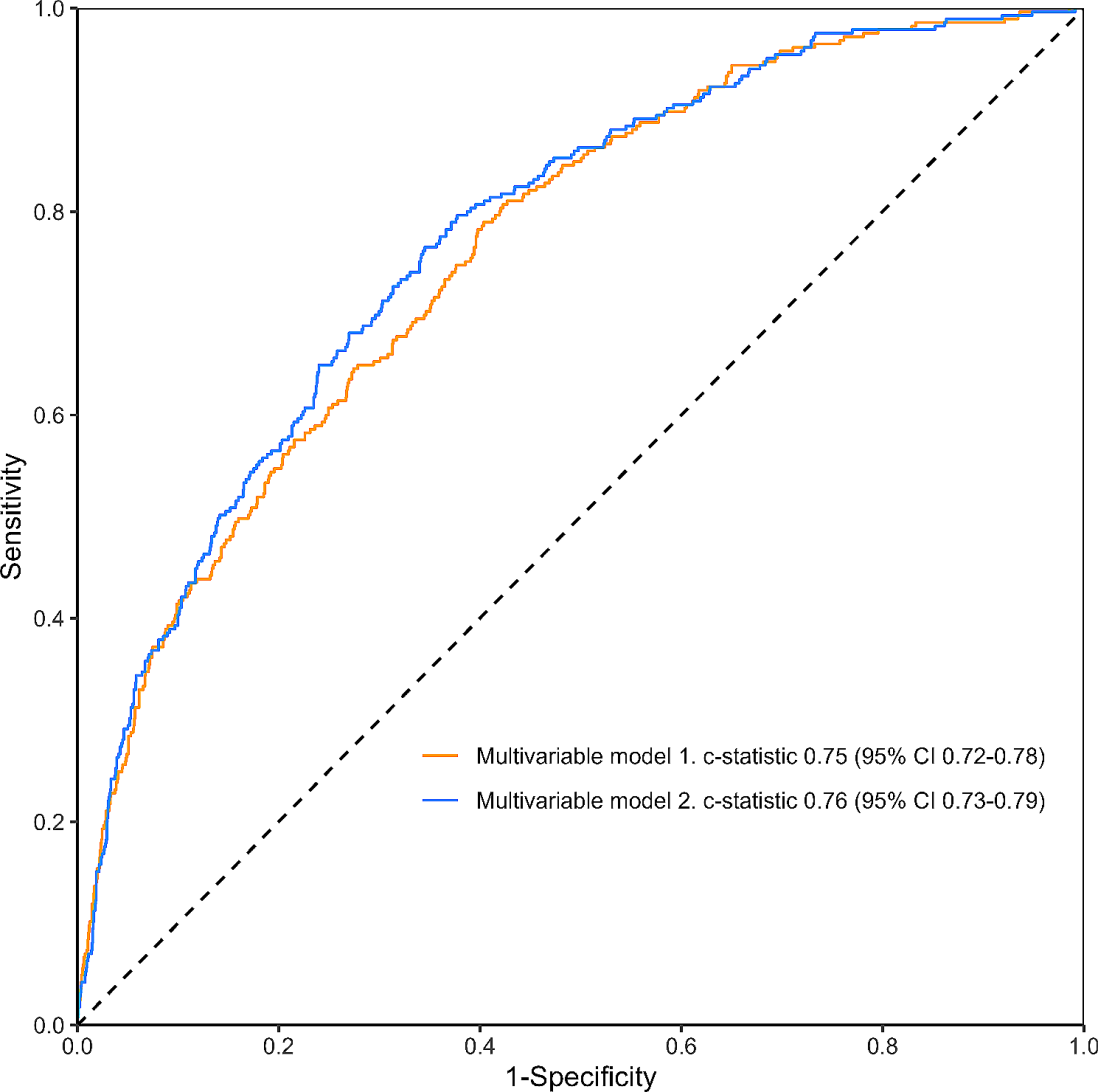
Receiver operating characteristics curves of multivariable logistic models with (Model 1) and without (Model 2) early postoperative fever

Figure 3 presents the receiver operating characteristic curves of multivariable Models 1 and 2. Detailed results of multivariable Model 1 are presented in Supplementary Table S2. The c-statistics were 0.75 (95% CI, 0.72–0.78) in Model 1 and 0.76 (95% CI, 0.73–0.79) in Model 2. The discriminative performance of Model 2 was significantly higher than that of Model 1 (difference in c-statistic, 0.02; 95% CI, 0.00–0.03; DeLong’s test, *P* = 0.005), indicating that the addition of early postoperative fever improved the predictive power of the model. Sensitivity, specificity, positive predictive value, negative predictive value, and accuracy for Models 1 and 2 are available in Supplementary Table S3.

## Discussion

This study explored the association between early postoperative fever and the occurrence of CR-POPF in patients who had undergone pancreaticoduodenectomy. Our results revealed a significant association between early postoperative fever and CR-POPF and identified early postoperative fever as a risk factor for CR-POPF. Furthermore, incorporating early postoperative fever into the well-established predictive factors for CR-POPF led to a notable improvement in the predictive power.

Previous studies have reported an association between postoperative fever and leakage at the site of the gastrointestinal anastomosis. A study of patients undergoing laparoscopic low anterior resection for rectal cancer identified fever on postoperative day 3 as an early predictive indicator of anastomotic leakage [20]. Another study in patients undergoing nondiverted large bowel resection found that fever on postoperative day 2 was associated with a higher risk of anastomotic leakage [21]. A retrospective analysis of computed tomography findings in patients undergoing gastric cancer surgery also found postoperative fever to be predictive of anastomotic leakage [22]. However, while postoperative fever has been reported as a common clinical symptom of POPF in previous studies [23, 24], the specific relationship between early postoperative fever and CR-POPF has not been previously explored. A systematic review identified postoperative fever as one of the most reliable indicators for the early recognition of CR-POPF [25]. However, this conclusion was based on only two studies, which included a total of 321 patients and did not specifically focus on postoperative fever. Several mechanisms may explain the association between early postoperative fever and CR-POPF. First, early postoperative fever may indicate a local inflammatory response, which can be triggered by factors such as exposure to pancreatic fluid, immune reactions, or other related complications [26]. Second, as suggested by our findings, infected POPF is commonly observed following pancreatoduodenectomy, often manifesting as early postoperative fever. Finally, fever itself can serve as a contributor to CR-POPF through pathophysiological mechanisms, including direct cellular damage and stimulation of cytokines and inflammatory responses [27]. Our study fills this gap by providing empirical evidence for this association.

The results of our study also demonstrated the predictive power of early postoperative fever when combined with previously identified predictors of CR-POPF. The addition of early postoperative fever to the multivariable model resulted in a significant, although small, improvement in predictive power. These results are likely because early postoperative fever is relatively common in pancreaticobiliary surgery [28], and the causes of early postoperative fever are highly diverse and expand beyond CR-POPF. Moreover, since CR-POPF arises from multiple factors, the addition of early postoperative fever to a predictive model that already incorporates well-known factors associated with its occurrence likely would not have resulted in a clinically significant enhancement of predictive power. Nevertheless, considering the significant association between early postoperative fever and the occurrence of CR-POPF, clinicians should contemplate the possibility of CR-POPF in patients presenting with early postoperative fever. In patients at high risk of CR-POPF, vigilant body temperature monitoring may be beneficial for the detection of early postoperative fever, enabling timely intervention through methods such as blood culture or computed tomography scans [29, 30]. Indeed, according to a recent large-scale randomized trial, implementation of algorithms that incorporate body temperature monitoring for early identification and management of postoperative complications after pancreatic resection can significantly reduce the risk of mortality by approximately 50% [31].

Our results revealed that early postoperative fever was significantly associated not only with CR-POPF, but also with postoperative pulmonary complications and wound complications. These associations were the probable causes of increased hospital stay. This result contradicts a recent study which reported that early postoperative fever was not associated with postoperative outcomes after upper abdominal surgery [28]. The clinical significance of early postoperative fever may, therefore, vary depending on the type of surgery. Early postoperative fever remains a meaningful indicator of potential complications in patients undergoing pancreaticoduodenectomy, warranting efforts to investigate its causes in such patients.

Our study also identified other risk factors for CR-POPF, including male sex, higher BMI, and higher fistula risk score. Conversely, undergoing surgery in the later study period was associated with a lower risk of developing CR-POPF. These findings are in accordance with the existing literature and illustrate the multifactorial nature of CR-POPF. In addition, severe intraoperative hypothermia, which we previously identified as a risk factor for CR-POPF [15], maintained a significant association even in the presence of additional covariates.

The strength of our study lies in its robust methodology, including using propensity score to balance the distribution of covariates between the early postoperative fever and no early postoperative fever groups. This approach enhanced the internal validity of the findings. However, this study has several limitations. First, it is a retrospective cohort study. Although significant efforts were made to adjust for previously identified risk factors for CR-POPF and to validate the results through multivariable logistic regression analyses, potential residual confounding bias cannot be entirely ruled out. However, the estimated E-value suggests that only unmeasured confounders with a strong association with both early postoperative fever and CR-POPF could explain the reported results, which could indicate the robustness of our findings. Additionally, while we were unable to include non-opioid analgesics with antipyretic effects in the analysis owing to the difficulty in accurately determining the temporal relationship between the administration of these medications and the onset of postoperative fever, in our previous research, the OR for the association between postoperative non-steroidal anti-inflammatory drugs (NSAIDs) and CR-POPF was 1.24 (95% CI: 1.05–1.47) [32], which was lower than the E-value assessed in this study. This finding suggests that the administration of postoperative NSAIDs is unlikely to negate the significance of the association between early postoperative fever and CR-POPF. Acetaminophen, another analgesic with antipyretic effects, was not used as a routine analgesic at our institution during the study period but only as an antipyretic. Therefore, it was not included in the analysis. Second, this study was conducted in a single-center setting with high case volume, which may limit the generalizability of the findings to other institutions with different clinical settings. However, pancreaticoduodenectomy is performed at our center by highly skilled surgeons, which likely minimizes potential surgeon-related variation. Additionally, approximately 90% of the patients analyzed in our study underwent open surgery. The present findings should be validated in hospitals that primarily use laparoscopic or robotic techniques, which are increasingly being implemented [33, 34]. Third, because our purpose was to investigate the association between early postoperative fever and CR-POPF, we limited the scope of the investigation to fever occurring in the first 48 h after surgery. Therefore, subsequent research is required to comprehensively address postoperative fever over an extended period. In addition, although postoperative fever can occur repeatedly, we did not consider this in our study. A recent study involving patients undergoing liver resection identified the occurrence of fever after postoperative day 2 and fever recurrence as significant risk factors for postoperative febrile infectious complications [35]. The same risk factors were identified as predictors of infectious febrile complications in elderly patients undergoing hemiarthroplasty [36]. Further research incorporating a continuous body temperature monitoring device for early detection of fever is needed to assess the temporal pattern of postoperative fever in patients undergoing pancreaticoduodenectomy and to evaluate its prognostic value to determine whether additional diagnostic workup is necessary. Fourth, fever may be both a causative factor and a resultant symptom, further complicating the interpretation of our findings. We acknowledge the limitations in suggesting interventions to prevent CR-POPF in patients who develop early postoperative fever. Additionally, given the retrospective nature of this study, we were only able to identify a significant association between early postoperative fever and the occurrence of CR-POPF without establishing a causal relationship between the two variables. Finally, although we proposed a potential mechanism by which early postoperative fever may contribute to CR-POPF, we could not determine whether the use of antipyretics to mitigate early postoperative fever decreased the incidence of CR-POPF. It is possible that the patients who received antipyretic treatment during the initial stages of fever development were classified into the no early postoperative fever group. Future prospective, randomized studies are warranted to investigate the potential effect of fever control on early detection of CR-POPF without masking signs of infection.

## Conclusions

In conclusion, this study identified a significant association between early postoperative fever and the occurrence of CR-POPF in patients undergoing pancreaticoduodenectomy. Although early postoperative fever was found to be a risk factor for CR-POPF, its high prevalence and small predictive power raise questions about its potential clinical application. However, this study also revealed associations between early postoperative fever and other postoperative outcomes. Further studies are required to elucidate the clinical significance of early postoperative fever and its potential application in postoperative care following pancreaticoduodenectomy.

### Electronic supplementary material

Below is the link to the electronic supplementary material.

Supplementary Material 1

## Data Availability

The datasets generated and analyzed during the current study are available from the corresponding author on reasonable request.

## Abbreviations

ASA: American Society of Anesthesiologists
ASD: absolute standardized mean difference
BMI: body mass index
CBD: common bile duct
CI: confidence interval
CR-POPF: clinically relevant postoperative pancreatic fistula
EPF: early postoperative fever
IRB: Institutional Review Board
NSAIDS: non-steroidal anti-inflammatory drugs
OR: odds ratios
POPF: postoperative pancreatic fistula
PPPD: pylorus-preserving pancreatoduodenectomy
sIPTW: stabilized inverse probability of treatment weighting
SUPREME: Seoul National University Hospital Patient Research Environment
