# Supplementary material for: Early postoperative fever as a predictor of pancreatic fistula after pancreaticoduodenectomy: a single-center retrospective observational study
Source: BMC Surg. 2024 Aug 12;24:229. doi: 10.1186/s12893-024-02521-0 (PMC11318233; doi:10.1186/s12893-024-02521-0)
Supplement: Supplementary file 1 — Supplementary Material 1 [file 12893_2024_2521_MOESM1_ESM.docx]

**Supplementary Material**

**Early Postoperative Fever as a Predictor of Pancreatic Fistula After Pancreaticoduodenectomy: A Single-Center Retrospective Observational Study**

Jae-Woo Ju, MD^1,2^, Hwan Suk Jang, MD^1^, Mirang Lee, MD^3^, Ho-Jin Lee, MD, PhD^1,2^, WooilKwon, MD, PhD^3,4^, and Jin‑Young Jang, MD, PhD^3,4^

**Author affiliations:**

^1^ Department of Anesthesiology and Pain Medicine, Seoul National University Hospital, Seoul, Republic of Korea

^2^ Department of Anesthesiology and Pain Medicine, Seoul National University College of Medicine, Seoul, Republic of Korea

^3^ Department of Surgery, Seoul National University Hospital, Seoul, Republic of Korea

^4^ Department of Surgery, Seoul National University College of Medicine, Seoul, Republic of Korea

**Corresponding author:**

Ho-Jin Lee, MD, PhD

Department of Anesthesiology and Pain Medicine, Seoul National University College of medicine Seoul National University Hospital, 101 Daehak-ro, Jongno-gu, Seoul 03080, Republic of Korea

Phone: 82-2-2072-2467

FAX: 82-2-747-8363

E-mail: [hjpainfree@snu.ac.kr](mailto:hjpainfree@snu.ac.kr)

**Table S1.** Comparison of other clinical outcomes after pancreaticoduodenectomy between patients with or without early postoperative fever (EPF)

|  | EPF group  (n=909) | No EPF group  (n=1088) | P-value |
| --- | --- | --- | --- |
| Infectious complications | 133 (14.6%) | 117 (10.8%) | 0.009 |
| Infected postoperative pancreatic fistula | 96 (10.6%) | 102 (9.4%) | 0.377 |
| Intra-abdominal abscess | 25 (2.8%) | 10 (0.9%) | 0.002 |
| Superficial/deep surgical site infection | 7 (0.8%) | 4 (0.4%) | 0.226 |
| Pneumonia | 6 (0.7%) | 3 (0.3%) | 0.315 |
| Phlebitis | 0 (0%) | 1 (0.1%) | >0.999 |
| Urinary tract infection | 2 (0.2%) | 1 (0.1%) | 0.594 |
| Sepsis | 7 (0.8%) | 1 (0.1%) | 0.027 |
| Culture test performed | 337 (37.1%) | 210 (19.3%) | <0.001 |
| Bacterial growth on culture test | 192 (21.1%) | 101 (9.3%) | <0.001 |
| Atelectasis | 320 (35.2%) | 326 (30%) | 0.013 |
| Serum C-reactive protein (mg/L) on postoperative day 1^a^ | 10.1 (6.3–14.7) | 7.4 (5.0–10.5) | <0.001 |

The values are presented as the median (interquartile range) or numbers (proportion).

^a^1230 missing values

**Table S2.** Multivariable logistic regression model incorporating preoperative and intraoperative variables for clinically-relevant pancreatic fistula after pancreaticoduodenectomy

|  | Multivariable | |
| --- | --- | --- |
|  | Adjusted OR  (95% CI) | *P*-value |
| Male (vs. female) | 1.81 (1.32–2.48) | <0.001 |
| Age, years | 1.01 (0.99–1.02) | 0.240 |
| Body mass index, kg/m² | 1.10 (1.06–1.15) | <0.001 |
| Current smoker | 0.87 (0.59–1.28) | 0.477 |
| Neoadjuvant chemotherapy | 0.25 (0.06–1.06) | 0.061 |
| Neoadjuvant radiotherapy | 0.88 (0.07–10.35) | 0.919 |
| Preoperative biliary drainage | 1.23 (0.89–1.69) | 0.216 |
| Pathology |  |  |
| Pancreatic cancer | Reference |  |
| Distal CBD cancer | 2.97 (1.91–4.62) | <0.001 |
| Ampulla of Vater cancer | 1.78 (1.14–2.79) | 0.012 |
| Duodenal cancer | 1.59 (0.67–3.81) | 0.296 |
| Neuroendocrine tumor | 2.41 (1.39–4.17) | 0.002 |
| Benign diseases | 1.74 (0.90–3.37) | 0.099 |
| Others | 1.76 (0.88–3.53) | 0.110 |
| Preoperative temperature, ℃ | 0.93 (0.61–1.40) | 0.714 |
| PPPD (vs. Whipple's operation) | 1.16 (0.80–1.68) | 0.435 |
| Robot-assisted (vs. open) | 1.10 (0.63–1.94) | 0.738 |
| Trans-anastomotic pancreatic ductal stent | 2.05 (0.89–4.73) | 0.092 |
| Fistula risk score (0-10) | 1.23 (1.12–1.34) | <0.001 |
| Operation time, hour | 0.99 (0.87–1.13) | 0.872 |
| Estimated blood loss, every 100 ml | 0.97 (0.93–1.01) | 0.172 |
| Intraoperative colloid, every 100 ml | 1.00 (0.95–1.04) | 0.887 |
| Intraoperative crystalloid, every 100 ml | 1.01 (0.99–1.03) | 0.196 |
| Intraoperative transfusion | 0.88 (0.55–1.41) | 0.596 |
| Intraoperative severe hypothermia | 1.72 (1.03–2.87) | 0.040 |
| Year of surgery |  |  |
| 2007-2010 | Reference |  |
| 2011-2013 | 0.98 (0.68–1.41) | 0.899 |
| 2014-2016 | 0.33 (0.21–0.50) | <0.001 |
| 2017-2019 | 0.35 (0.22–0.55) | <0.001 |

OR, odds ratio; CI, confidence interval; CBD, common bile duct; PPPD, pylorus-preserving pancreatoduodenectomy.

**Table S3**. Sensitivity, specificity, positive predictive value, negative predictive value, and accuracy of the Models 1 and 2.

|  | Sensitivity | Specificity | Positive predictive value | Negative predictive value | Accuracy |
| --- | --- | --- | --- | --- | --- |
| Model 1 | 0.789 | 0.567 | 0.246 | 0.944 | 0.598 |
| Model 2 | 0.765 | 0.655 | 0.269 | 0.944 | 0.658 |
